# Expected Liking of and Emotional Responses to Alternative Protein Burger Patties Among a Convenience Sample of UK Meat Eaters

**DOI:** 10.3390/foods15091538

**Published:** 2026-04-29

**Authors:** Yuchen Zhang, Margaret Thibodeau, Rebecca Ford, Qian Yang

**Affiliations:** Sensory Science Centre, Division of Food, Nutrition & Dietetics, School of Biosciences, University of Nottingham, Sutton Bonington Campus, Loughborough LE12 5RD, UKmargaret.thibodeau@nottingham.ac.uk (M.T.); r.ford@nottingham.ac.uk (R.F.)

**Keywords:** protein alternatives, emotional responses, information effect, cluster analysis, food neophobia, food technology neophobia, food disgust

## Abstract

Increasing the adoption of protein alternatives could be one of the solutions for improving the sustainability of our current food system. A convenience sample of 574 UK meat eaters completed an online survey incorporating a written shopping scenario (71% female, mean age: 31.8 years). A subset of participants (*n* = 297) also viewed a video describing the environmental impacts of protein sources and the lab-grown meat production process. Participants imagined selecting burger patties (plant-based, edible insect, lab-grown beef, or conventional beef) from a supermarket shelf and completed measures of expected liking, emotional responses, choice, and food-avoidance traits (food neophobia, food technology neophobia, and food disgust). The beef burger patty was selected the most frequently (63%), associated with positive emotions, and most liked. Plant-based (19%) and lab-grown beef patties (15%) were chosen more often than edible insect patties (2%), reflecting differences in expected liking and an increasing tendency for them to be associated with negative emotions. Trait-based clustering identified four groups (food explorers, novel/disgust fearers, food tech fearers, and everything fearers), each with distinct liking, emotion, and choice patterns for the products. Food explorers appeared to be the most likely early adopters of protein alternatives, especially when compared to everything fearers. Participants who viewed the informational video were more likely to select a protein alternative, although the effect on liking was limited. These findings demonstrate that emotional responses and food-related personal traits play a central role in shaping consumer acceptance of protein alternatives, suggesting that strategies to promote sustainable protein consumption should be tailored to specific consumer segments and product types.

## 1. Introduction

The current global food system and consumer food consumption patterns are unsustainable for human and planetary health [1]. With the challenges of population growth, ageing, and climate change, there is growing demand for a sustainable food system transition. The United Nations (UN) warned that ‘Unless we act now, the 2030 Agenda will become an epitaph for a world that might have been’ [2]. One pathway to supporting the UN Sustainable Development Goals is the promotion of sustainable dietary patterns. It is well known that red meat, such as beef, generates higher greenhouse gas emissions than plant-based alternatives [3,4]. Accordingly, reducing red meat consumption and increasing the intake of sustainable protein alternatives (e.g., plant-based protein, lab-grown meat, mycoprotein, algae, insects, and hybrid meats [5]) has been identified as a potential strategy to facilitate the transition towards a more sustainable food system [6,7]. With the increasing global demand for protein, there is a need for a wide range of protein sources that are accessible and accepted by consumers.

Despite their environmental benefits, consumers are often reluctant to adopt protein alternatives, especially when they are unfamiliar or novel [8]. Early plant-based meat alternatives were primarily derived from processed plant ingredients, such as soybeans, wheat gluten, and pulses [9]. These included products such as tofu, which were not originally designed as meat analogues and therefore do not replicate the sensory attributes of meat. In turn, this may limit their adoption as protein alternatives in cultural contexts where familiarity is low [8]. Advances in food science and biotechnology led to the development of second-generation plant-based meat alternatives [9]. These products incorporate more intensive processing technologies such as thermal extrusion, fermentation, and protein cross-linking, as well as the addition of flavourings, with the aim of more closely mimicking the texture and flavour of conventional meat [9,10,11], thereby helping to reduce the familiarity gap when compared with conventional meat products.

Edible insects have been consumed historically across many cultures in Africa and Asia; however, they are generally considered a novel protein source that often elicits disgust among Western consumers [12]. In the UK, several edible insect species (e.g., yellow mealworm, house cricket, branded cricket, and black soldier fly) have been approved for human consumption by the Food Standard Agency [13]. Despite the regulatory approval, the market for edible insect products in the UK remains limited. Consumer acceptance of edible insects is shaped by cultural norms, food neophobia, and disgust sensitivity, which can limit consumer willingness to try or consume edible insect food products [14]. Consequently, edible insects face greater acceptance challenges than other alternative proteins that more closely resemble familiar foods.

Lab-grown meat, also known as cultured meat or cultivated meat, has the potential to closely replicate the nutritional and sensory properties of conventional meat [15,16]. It is produced from animal cells taken from living animals and then grown in a nutrient-rich medium in a controlled environment [17]. As real animal cells are used, it may offer consumers a more environmentally friendly alternative that feels less novel, as both the taste and preparation methods are expected to be similar to those of conventional meat [18]. While lab-grown meat has not yet been approved for human consumption in the UK, a significant regulatory milestone was reached in 2024 when lab-grown meat was approved for use in pet food [19]. This early-stage approval signals both regulatory openness and growing commercial momentum, suggesting potential future applications for human consumption. However, as lab-grown meat production differs substantially from traditional meat production, the novelty of the production process may present challenges for consumer acceptance [20,21]. As public and private investment in the lab-grown meat sector continues to grow, understanding consumer expectations will become increasingly important.

Studying personal traits associated with the acceptance of novel protein alternatives can provide insights into why consumers have traditionally been reluctant to adopt them [22,23]. Consumers are not a homogeneous group; therefore, it may be possible to identify segments that are more likely to transition away from conventional meat at an earlier stage. It is also plausible that different consumer groups may follow distinct transition pathways by selecting different types of alternative proteins. Some of the commonly studied personal traits include food neophobia, food technology neophobia, and food disgust sensitivity. Together, these traits capture avoidance-related responses to unfamiliar foods and food production processes, which are particularly relevant in the context of novel and emerging protein alternatives. Food neophobia is defined as an aversion to trying new or novel foods, and can be measured using the food neophobia scale (FNS) [24,25]. Consumers with high levels of food neophobia have been shown to have lower acceptability of a range of protein alternatives, such as plant-based protein, lab-grown meat, and edible insects [26,27,28]. The food technology neophobia scale (FTNS) was developed by Cox and Evans [29] to measure reluctance towards new food technologies. Recent research found that individuals with high food technology neophobia are less willing to try new foods produced by novel technologies, especially lab-grown meat [30,31]. Food-related disgust is an emotion that prompts the rejection of food and is primarily aimed at avoiding the consumption of substances that may be harmful or contaminated with pathogens [32,33]. A person’s food disgust sensitivity can be measured using the food disgust scale [32,33]. Niimi et al. [34] found that individuals that experience more disgust in response to food and food-related situations (i.e., higher food disgust sensitivity) were less likely to accept edible insects. Although these personal traits have been examined across multiple studies, they are typically treated independently, despite evidence that they are correlated [32,33,35]. Moreover, as individuals represent combinations of these traits, studying them in isolation may limit the practical applicability of findings. As a result, there is currently a lack of understanding of how these traits interact to influence consumer acceptance of protein alternatives. Therefore, cluster analysis could provide a more holistic understanding of consumer segments based on these personal traits rather than isolated variables. This study extends existing work by applying a cluster-based approach to examine how combinations of food technology neophobia, food neophobia, and food disgust sensitivity relate to consumer responses to protein alternatives, therefore providing more actionable insights into individual differences in alternative protein acceptance and potential targets for tailored communication strategies.

Additionally, sharing information as an intervention strategy to enhance consumer acceptance of protein alternatives has been a common focus in research [36,37,38,39,40,41]. It has been shown that consumer liking of protein alternatives can be significantly increased by presenting information on the front of food packaging, incorporating text and imagery [36,39,41], or utilising food-labelling strategies [37]. In addition, the environmental benefits of concise texts and carbon labelling have been shown to positively impact consumer acceptance as measured by overall liking and purchase intention [37,42,43]. However, there are also studies suggesting that the impact of sharing information is limited. Grasso, Rondoni, Bari, Smith, and Mansilla [39] indicated that food ingredient information did not significantly impact the overall liking of a hybrid burger (60% beef and 40% vegetables). As personal traits are known to influence expected liking of protein alternatives, it is plausible that information provision similarly exerts segment-specific effects that are not captured when consumers are treated as a homogeneous group [5,44].

Therefore, this study aimed to explore expected consumer responses to both novel protein sources (lab-grown meat and edible insects), established alternatives (plant-based meat), and conventional beef. Burger patties were selected because conventional beef burgers are widely consumed, and the burger format is a commonly available and comparable product format across both meat and meat-alternative categories. More specifically, this study aimed (1) to investigate UK consumers’ expected liking, purchase intention, and emotional responses towards the burger patties; (2) to conduct a preliminary and exploratory cluster analysis to determine if the participants can be clustered based on three food-avoidance-related personal traits (food neophobia, food technology neophobia, and food disgust); (3) to examine if these clusters differ in their responses to the four protein types; and (4) to exploratorily evaluate the impact of information sharing (environmental impact and lab-grown meat production process) on the participant’s responses. Based on previous research, it was hypothesised that expected liking, purchase intention, and emotional responses would differ across protein sources, with conventional beef burger patties rated most favourably, and edible insect patties rated least favourably. Second, it was hypothesised that food neophobia, food technology neophobia, and disgust would be negatively associated with the liking of the protein alternatives. Third, it was hypothesised that providing environmental information would positively influence consumer responses towards the alternative protein sources. Collectively, this study is novel in its integrated examination of product type, information provision, and trait-based consumer segmentation across multiple protein sources within a single experimental framework. Despite using a convenience sample, the study provides exploratory insights that are critical for informing future product design, communication strategies, and the direction of future follow-up studies.

## 2. Materials and Methods

### 2.1. Participants, Survey Structure, and Video Sharing Conditions

A convenience sample of participants was recruited (1) via email and poster advertisement across the University of Nottingham’s campus and (2) via the Prolific platform (Prolific, London, UK). Participants were eligible for the study if they were 18 years old or older, had lived in the UK for at least 3 months, and self-reported as meat eaters (omnivores and flexitarian). As this study was collecting responses to conventional beef products, non-meat eaters (vegetarians, vegans, and pescatarians) were excluded from the study. The study received a favourable ethical opinion from the University of Nottingham’s School of Bioscience Research Ethics Committee (SBREC202223026FEO).

A total of 827 participants consented to take part in the study. However, 209 participants were excluded as they closed the survey before answering all the questions (25.2%). The majority of them were recruited via email or poster at the university (*n* = 199, 95.2%). Participants (*n* = 27) were also excluded for completing the questionnaire too quickly if their completion time was less than 60% of the median response time within their respective video exposure conditions, which were 11 min for participants who watched the video and 7 min for those who did not [45]. Finally, 23 participants were excluded from the study as post-data collection checks indicated that they did not meet the eligibility criteria of the study. Thus, a convenience sample of 574 participants (33.6 ± 11.9 years, 408 female and 166 male) was retained for the analysis. Full demographic data (age, gender, education, diet) is reported in Table A1 and Table A2.

**Figure 1:**
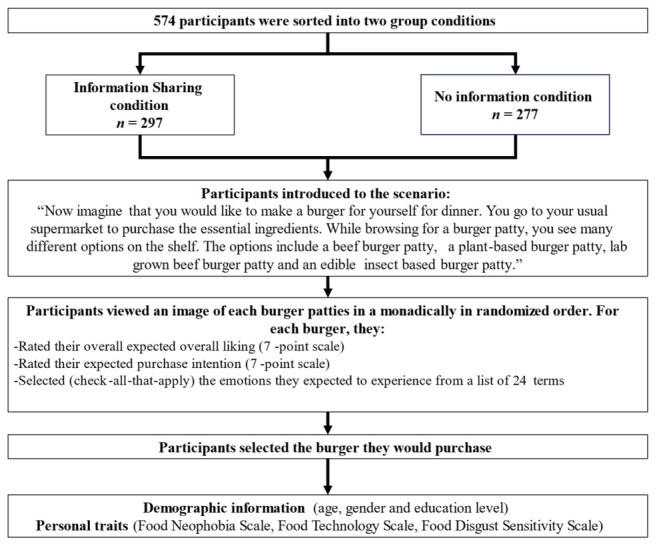
Survey structure flowchart (*n* = 574).

Two separate but structurally identical 15 min online surveys were run concurrently to allocate participants to one of two experimental conditions. Participants allocated to the information sharing condition (*n* = 297) began the survey by watching an environmental video, whereas those in the no information condition (*n* = 277) proceeded directly to the survey without viewing the video. For university-recruited participants, advertisement materials were identical across conditions except for the survey link, which directed participants to one of the two versions. Similarly, Prolific participants were recruited using identical survey descriptions for both conditions. Consequently, all participants were allocated to a condition without prior knowledge of which version of the survey they would receive. Although this procedure resulted in slightly unequal numbers of participants across conditions, it enabled counterbalancing across recruitment sources. Figure 1 provides an overview of the survey flow and participant allocation across study stages.

The environmental video outlined key contributors to the environmental impact of food systems, with a focus on greenhouse gas emissions derived from life-cycle assessment data. The video was previously used in Ford et al. [46], a study that captured consumer responses towards ‘precision-fermented’ yoghurt. The video explained that global food production accounts for approximately 26% of anthropogenic greenhouse gas emissions, with around half attributable to meat production. The video compared emissions across food categories, highlighting that beef and lamb have substantially higher carbon footprints than poultry, plant-based foods, and dairy alternatives. The video also introduced emerging sustainable food technologies, including cellular agriculture, which produces animal-derived foods without raising or slaughtering animals. It described the basic processes for producing cultivated meat, where animal cells are sampled and grown into muscle tissue, and precision-fermented dairy, where milk proteins are generated by engineered microorganisms. Evidence from recent life-cycle assessments was presented, indicating that cultivated beef may reduce emissions by up to 87%, and precision-fermented dairy may reduce emissions by 35–65%, compared with their conventional counterparts. The video concluded by noting additional future food options such as edible insects, which have significantly lower emissions than traditional livestock, and emphasised the broader challenge of achieving sustainable global food production.

### 2.2. Shopping Scenario and Products

To explore the impact of burger patty type on consumer responses, a written scenario was used to simulate the context, as follows: ‘Now imagine that you would like to make a burger for yourself for dinner. You go to your usual supermarket to purchase the essential ingredients. While browsing for a burger patty, you see many different options on the shelf. The options include a beef burger patty, a plant-based burger patty, a lab-grown beef burger patty, and an edible insect-based patty.’ Below the written scenario, participants were also able to see an image of all four burger packages (Table 1). The term ‘lab-grown’ meat is used throughout this paper, although alternative terms such as ‘cultured meat’ or ‘cultivated meat’ are increasingly used in academic and industry contexts. ‘Lab-grown’ meat was used in this study because it is more widely recognised by the general public and was used more frequently in the media at the time of the data collection [15,21].

The packaging was designed to simulate a supermarket environment and to convince participants that alternative products were meat-like, with similar sensory qualities to a conventional burger. A background image of a person holding a tray with two burger patties at a supermarket was downloaded from Shutterstock Inc. (New York, NY, USA). The packaging format and design were kept largely consistent for all four products. However, the top portion of the label was customised for each burger patty type by changing the name and adding an image of the main protein source. Raw beef patties were selected for the top of the label of both beef products (conventional beef and lab-grown patties) on the assumption that when lab-grown beef is available, it will mimic the sensory properties and appearance of conventional beef. For the edible insect burger patty, an image of raw buffalo worms was used, and for the plant-based burger patty, an image of peas was displayed.

After reviewing the context, participants evaluated each of the burger patties monadically in randomised order. Participants were asked to imagine picking up each product from the supermarket shelf based on the image provided with a short, written description (Table 1). Next, the participants rated their expected overall liking (7-point hedonic scale), expected purchase intention (7-point scale), and emotional responses (24-item plant-based specific emotion lexicon using “check all that apply” [47]). The emotion terms were presented in a balanced randomised order across participants but kept consistent for each participant. Capturing consumers’ emotional responses to different protein alternatives could provide valuable information, as emotional responses have been shown to offer additional insights compared to hedonic liking alone [43,48]. After participants had evaluated all products, they were asked to indicate ‘Which burger patty do you think you would like to purchase and take home?’.

### 2.3. Demographics and Personal Traits

Participants were asked to answer a range of demographic questions (e.g., age, gender, and education level). Next, using 7-point scales (‘Strongly disagree’ to ‘Strongly agree’) participants completed the FNS (10 items, score range 10–70 [24]) and the FTNS (13 items, score range 13–91 [29]). Participants also completed a modified version of the short FDS (all items except LCON1, 7 items, score range 7–42 [32,33]), using a 6-point scale from ‘Not disgusted (grossed out) at all’ to ‘Extremely disgusted (grossed out)’ [32,33]. Responses were summed to obtain three scores for each participant, one for each of the personal trait scales. All three personal traits had acceptable internal consistency based on Cronbach’s Alpha [49]: FNS (Cronbach’s alpha = 0.70), FTNS (Cronbach’s alpha = 0.80), and FDS (Cronbach’s alpha = 0.70). To better interpret and compare differences across the three personal traits, scores were z-score normalised to centre the data around the mean and standard deviation for easier comparison across scales [33]. As there are differences in the range of possible scores for each of the scales, this makes it easier to visually identify which segments scored higher or lower than average across the three traits.

### 2.4. Data Analysis

To address the study objectives, a series of statistical analyses was conducted. All data were analysed using XLSTAT [50]. A significant value of *p* < 0.05 was adopted for all hypothesis tests. The Sheskin procedure was selected as the post-hoc test for Cochran’s Q. For Chi-square contingency analyses, effect sizes were assessed using Cramér’s V, with values interpreted as follows: 0.00–0.10 is negligible, 0.10–0.20 is weak, 0.20–0.40 is moderate, 0.40–0.60 is relatively strong, 0.60–0.80 is strong, and 0.80–1.00 is very strong [51].

For ANOVA and mixed-model analyses, model assumptions were evaluated post hoc through inspection of residuals, and no substantive deviations from parametric assumptions were detected. Tukey’s HSD was used for post-hoc comparisons. Effect sizes for fixed effects were obtained using partial eta^2^ (*η_p_*^2^) and were interpreted as *η_p_*^2^ < 0.01 is ‘negligible’, 0.01 ≤ *η_p_*^2^ < 0.06 is ‘small’, 0.06 ≤ *η_p_*^2^ < 0.14 is ‘medium’, and *η_p_*^2^ ≥ 0.14 is ‘large’ [52]. Where appropriate, participant was included as a random factor to account for within-participant variability; random participant effects were not interpreted, while all fixed effects are fully reported. 

Seven-point scales were selected to measure liking and purchase intention, as no training is needed prior to use [53]. Although the data is technically ordinal, rating scales are commonly treated as continuous in behavioural and sensory research. Modelling studies indicate that scales with five or more response options can be analysed as continuous without substantively violating parametric assumptions [54]. Furthermore, in a review, Lim [53] suggest that these violations are also smaller in large sample sizes (*n* > 75). Thus, liking and purchase intention were treated as a continuous variable to allow for the use of parametric statistics, which are more powerful than their non-parametric equivalents.

To examine product differences, mixed model ANOVAs (fixed effect: burger patty type, random effect: participant) were performed to test for significant differences in the liking and purchase intention between the four burger patties. Pearson’s correlation was used to examine the correlation between liking and purchase intention. Correspondence analysis and Cochran’s Q tests were used to test whether differences between the four products existed for the citation proportion of the emotion terms. To correct for multiple comparisons in the Cochran’s Q tests (*n* = 24), the Bonferroni correction was used to calculate an adjusted alpha of 0.021 as the benchmark for significance.

**Figure A1:**
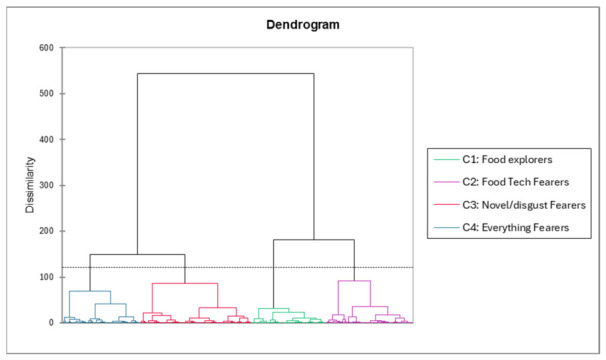
Dendrogram illustrating the agglomerative hierarchical clustering solution based on participants’ centered and scaled food neophobia, food technology neophobia, and food disgust scores, using Ward’s method with Euclidean distance. The dashed horizontal line indicates the cut-off height used to define the clustering solution, yielding four clusters (C1–C4).

Previous literature indicates that the FNS, FTNS, and FDS are related personal traits, a pattern that was confirmed in the present study using Pearson’s correlations (FNS & FTNS: r(2294) = 0.201, 95% CI [0.161–0.240], *p* < 0.001; FNS & FDS: r(2294) = 0.468, 95% CI [0.435–0.499], *p* < 0.001; FTNS & FDS: r(2294) = 0.155, 95% CI [0.115–0.195], *p* < 0.001). Rather than examining these traits in isolation, the present work aimed to identify participant groups characterised by combinations of these personal traits. Accordingly, cluster analysis was employed to group participants based on their joint FNS, FTNS, and FDS scores. Agglomerative hierarchical clustering using Ward’s method with Euclidean distance (centre and reduce) was performed based on participants’ FNS, FTNS, and FDS scores. A four-cluster solution was selected following visual inspection of the dendrogram (Figure A1), evaluation of cluster interpretability and face validity, and consideration of meaningful separation across FNS, FTNS, and FDS scores. One-way ANOVA was conducted to examine the differences between clusters on the FNS, FTNS, and FDS scores. Similarly, ANOVA was used to test for differences in mean age between the clusters. Finally, associations between the clusters and gender, education, and diet preference were tested using the Chi-square test.

A mixed-model ANOVA (fixed effect: burger patty type, personal trait cluster, burger patty type*personal trait cluster; random effect: participant (personal trait cluster)) was performed to test for differences in the liking of the burger patties. To account for unequal cluster sizes, emotion counts were converted to selection percentages by dividing the number of selections for each emotion by the total number of respondents within each cluster multiplied by 100. These percentage values then underwent principal component analysis to visualise the underlying structure of emotional profiles. A Chi-square test was used to test for an association between cluster and expected burger patty choice. However, the edible insect burger category was excluded from the contingency analysis due to low cell counts.

To investigate the impact of information sharing on expected liking, a mixed-model ANOVA (fixed effect: burger patty type, information sharing group, burger patty type*information sharing group; random effect: participant(information sharing group)) was also performed. Finally, a Chi-square test was used to test for an association between information sharing and expected burger patty choice.

## 3. Results

### 3.1. Overall Liking, Purchase Intention, and Emotional Responses

**Figure 2:**
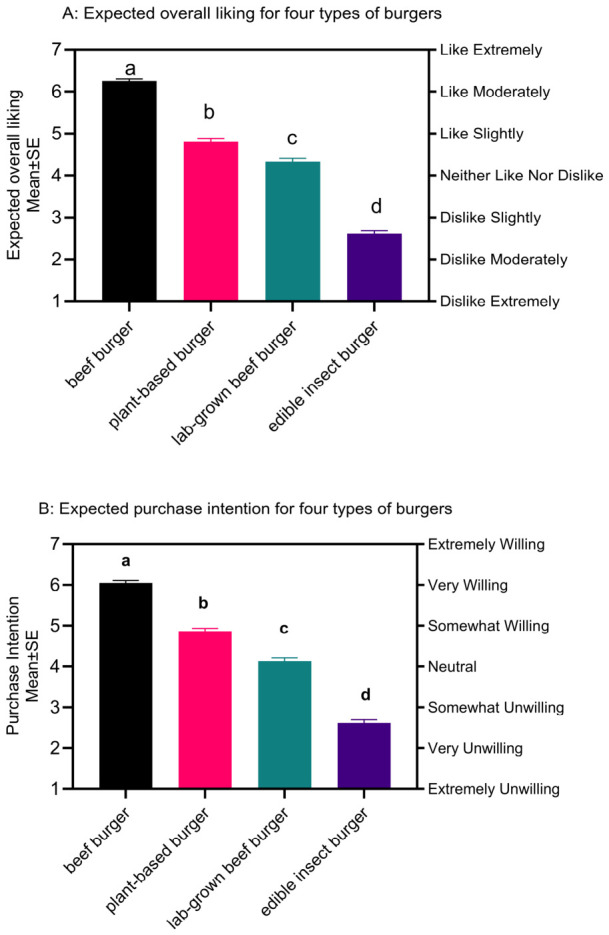
Mean expected overall liking ((**A**); dislike extremely = 1; like extremely = 7) and purchase intention ((**B**); 1 = extremely unwilling; 7 = extremely willing) of the four burger patty types (conventional beef, plant-based, lab-grown, and edible insect). Products that differ significantly in liking are indicated with different letters (*p* < 0.05, main effect, mixed-model ANOVA, Tukey’s HSD).

Mixed model ANOVA indicated a significant difference among the four burger patties (Figure 2A, (F(3,1719) = 581.3, *p* < 0.001, *η_p_*^2^ = 0.50). Post-hoc tests revealed that all four products had significantly different liking scores from each other. Overall, the conventional beef burger patty was most liked, with a mean liking ‘liked moderately’ (M = 6.25, 95% CI [6.16–6.35]). On average, the plant-based burger patty was ‘slightly liked’ (M = 4.80, 95% CI [4.67–4.93]), while the lab-grown beef burger patty (M = 4.33, 95% CI [4.18–4.49]) was closer to ‘neither liked nor disliked’. The edible insect burger patty had the lowest mean liking score (M = 2.61, 95% CI [2.47–2.76]), between ‘dislike slightly’ and ‘dislike moderately’.

Participants’ purchase intention for the burger patties were consistent with liking (Figure 2B), with participants being more willing to purchase the burgers that they expected to like. A mixed-model ANOVA confirmed that purchase intention for all the products was significantly different (F(3,1719) = 402, *p* < 0.001, *η_p_*^2^ = 0.44). As overall liking and overall purchase intention were highly correlated (r(2294) = 0.871, 95% CI [0.861–0.881], *p* < 0.001), no further analysis of the purchase intention data was performed.

**Figure 3:**
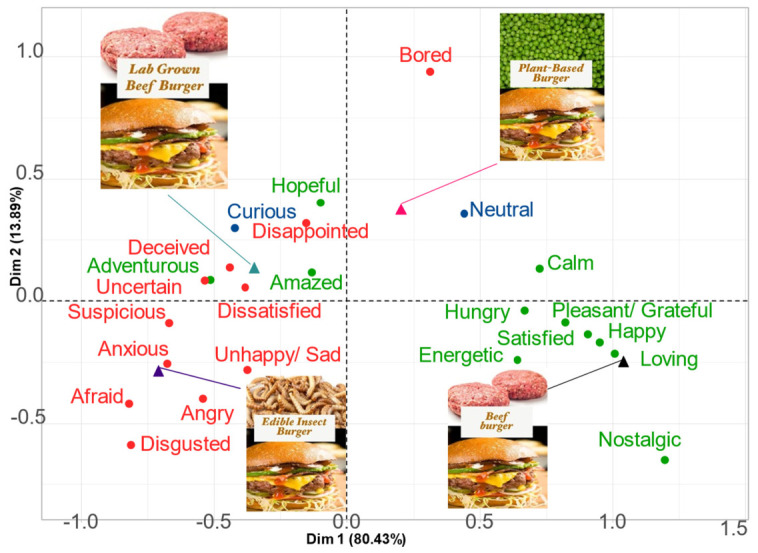
Overall emotional response evoked by the four burger patty types (conventional beef, plant-based, lab-grown beef, and edible insect). Emotions are classified into positive (*n* = 11, green), negative (*n* = 11, red), and unclassified (*n* = 2, blue) groups based on Orr, Giezenaar, Godfrey, and Hort [47].

For all the emotion terms, significant differences in the citation proportions were found between the burger patties (Cochran’s Q with Bonferroni correction, all *p* < 0.0001, Table A1). Thus, correspondence analysis was performed on the full lexicon (Figure 3). A clear separation between the four burger patty types can be seen, indicating that each product evoked a different emotional response. Dimension 1 explained 80.5% of the variation, mainly separating the emotions from positive to negative. Dimension 2 explained 13.9% of the variance and reflected a dominance-related dimension, distinguishing responses associated with a sense of agency/control from those reflecting more externally driven emotional reactions. The positive emotions (*satisfied*, *happy*, *pleasant/grateful*, *hungry*) and the unclassified emotion (*neutral*) were largely selected for the products that are currently available in the UK market, namely the conventional beef and plant-based burger patties. While for the positive emotions the citation proportion was higher for the conventional beef burger patty than the plant-based burger patty, the opposite trend was observed for *neutral*. The negative emotions were generally associated with products not currently available in UK supermarkets, such as lab-grown beef and edible insect burger patties. Some of these emotions were cited equally for both products (*uncertain*, *suspicious*, *adventurous*), while others were cited more frequently for the edible insect burger patty (*disgusted*, *anxious*, *afraid*). Participants also expected the lab-grown burger patty to evoke both negative (*deceived and uncertain*) and positive emotions (*hopeful and amazed*). This suggests that the lab-grown burger evoked heterogeneous emotional responses. Participants were more likely to select *hopeful* for the plant-based burger patty than for the conventional beef burger patty. Moreover, a significantly higher proportion of participants expected to be *dissatisfied* and *disappointed* by the edible insect and plant-based burger patties compared to the products made with beef. Finally, *curiosity*, an unclassified emotion, was selected most frequently for the lab-grown beef burger patty. *Curious* was also selected to a lesser extent for the edible insect and plant-based burger patties, but was generally not selected by participants in response to the conventional beef burger patty.

After evaluating the products monadically, participants were asked to select the burger patty type they would hypothetically purchase. Most participants (63.2%) selected the conventional beef burger patty, which is consistent with the fact that it scored highest for liking and was most closely associated with positive emotions. Also consistent with liking scores, the plant-based burger patty (19.3%) was the next most selected, followed by the lab-grown beef burger patty (15.0%) and the edible insect burger patty (2.4%).

### 3.2. Personal Trait Clusters

**Figure 4:**
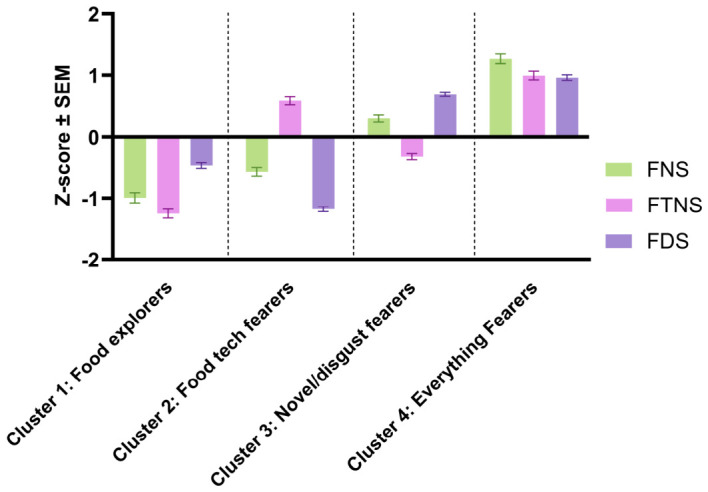
Mean z-scores (±SEM) for the food neophobia scale (FNS), the food technology neophobia scale (FTNS), and the food disgust scale (FDS) for each of the consumer clusters (food explorers (*n* = 122, 21%), everything fearers (*n* = 128, 22%), novel/disgust fearers (*n* = 182, 32%), food tech fearers (*n* = 142, 25%)).

Cluster analysis of raw scores for the FNS, FTNS, and FDS led to the identification of four consumer clusters, each with a distinct combination of personal traits. Figure 4 presents the mean standardised z-scores for each of the scales by consumer cluster. Mean z-scores above zero indicated that participants exhibited higher levels of these traits compared to the overall mean score with the whole study population, whereas a score below zero reflects lower levels. One-way ANOVAs confirmed that significant differences in FNS, FTNS and FDS scores were found between the clusters. The differences between the clusters are summarised below, with full details available in Table A2.

Cluster 1 (21%) had the lowest mean FNS and FTNS scores, with z-scores approximately one standard deviation below the mean. Thus, as Cluster 1 had the lowest levels of food and food technology neophobia, they can be considered the most open to trying new and unfamiliar foods, even if these foods are made with novel technologies. Furthermore, Cluster 1 also had the second lowest mean FDS scores, indicating that participants in Cluster 1 experienced relatively low levels of disgust in food and food-related situations. Therefore, Cluster 1 was named food explorers.

Cluster 2 (25%) had the second highest FTNS scores, but their mean FNS (second lowest) and FDS (lowest) scores were below average. Taken together, these results suggest that for Cluster 2, the personal trait that is most likely to impact their food-related behaviour is food technology neophobia. Thus, Cluster 2 was named food tech fearers.

Cluster 3 was the largest cluster (32%) and when compared to Cluster 2 showed the opposite trend. The mean scores for Cluster 3 were above average for the FNS and FDS (both second highest), but were lower for the FTNS (second lowest). This suggests that for Cluster 3, the inherent characteristics of foods themselves (e.g., novelty or ability to elicit disgust) rather than the processes used to produce them are more likely to impact their behaviour. Thus, they were named novel/disgust fearers.

Finally, Cluster 4 (22%) had the highest mean score for the FNS, FTNS, and FDS, with scores all one standard deviation above the mean. Thus, compared to the other clusters, Cluster 4 experiences the most food neophobia and food-related disgust, suggesting that they are likely more cautious or hesitant about all types of foods. As a result, Cluster 4 was named everything fearers.

A Chi-square test revealed a significant but weak association between personal trait cluster and dietary preference (*p* = 0.007, Cramér’s V = 0.15, Table A2). Novel/disgust fearers were more likely to identify as flexitarians (12.6%) and less likely to identify as omnivores (87.4%) than other clusters (flexitarians (3.9–4.9%) and omnivores (95.1–96.1%)). No significant differences between the clusters were found based on age (ANOVA, *p* = 0.287, Table A1), gender (Chi-square, *p* = 0.128, Table A2), or education level (*p* = 0.147, Table A2).

### 3.3. The Impact of Personal Trait Cluster on Consumer Responses

**Figure 5:**
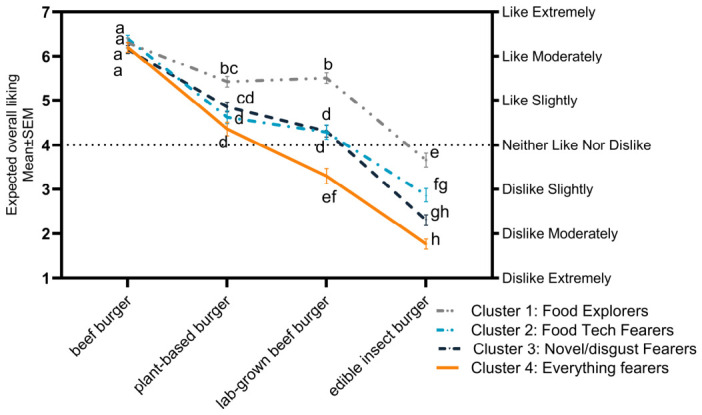
Interaction plot comparing the mean liking scores of participants in the personal trait cluster (food explorers, food tech fearers, novel/disgust fearers, and everything fearers) across the four burger patty types (conventional beef, plant-based, lab-grown beef, and edible insect). Significant differences in liking are indicated with different letters (*p* < 0.05, mixed-model ANOVA interaction, Tukey’s HSD).

A mixed-model ANOVA revealed a significant main effect of personal trait cluster on the overall expected liking of the burger patties (F(3,1710) = 48.6, *p* < 0.0001, *η_p_*^2^ = 0.11). When averaged across all the burger patty types, food explorers reported the highest overall liking scores and everything fearers had the lowest liking scores. Food tech fearers and novel/disgust fearers had liking scores between the other two clusters and did not differ from one another. However, this pattern was not consistent for all the burger patty types (Figure 5), as indicated by the significant interaction between burger patty type and personal trait cluster (F(9,1710) = 10.3, *p* < 0.0001, *η_p_*^2^ = 0.051). The expected liking of the conventional beef burger patty did not differ between the clusters, likely reflecting its widespread availability and familiarity. In contrast, between-cluster differences were observed for the remaining three burger patties, which are less commonly available and/or consumed. The food explorers expected to like each of these burgers more than the other clusters, with one exception: the plant-based burger patty, where no significant difference in expected liking was observed between food explorers and novel/disgust fearers. The most pronounced differences in expected liking were found in the lab-grown beef burger patty, with food explorers expecting to moderately like it and everything fearers expecting to dislike it slightly. While significant differences were found between clusters for the plant-based and edible insect burger patties, the difference was not as wide as for lab-grown beef.

**Figure 6:**
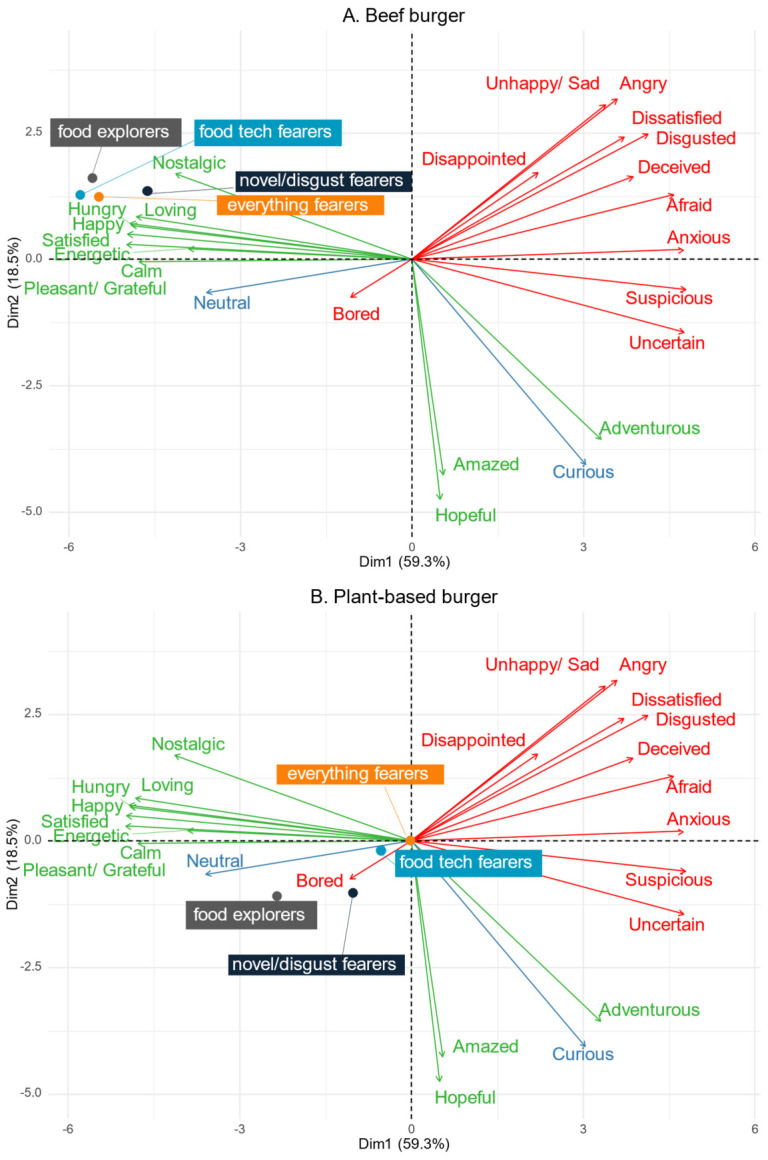

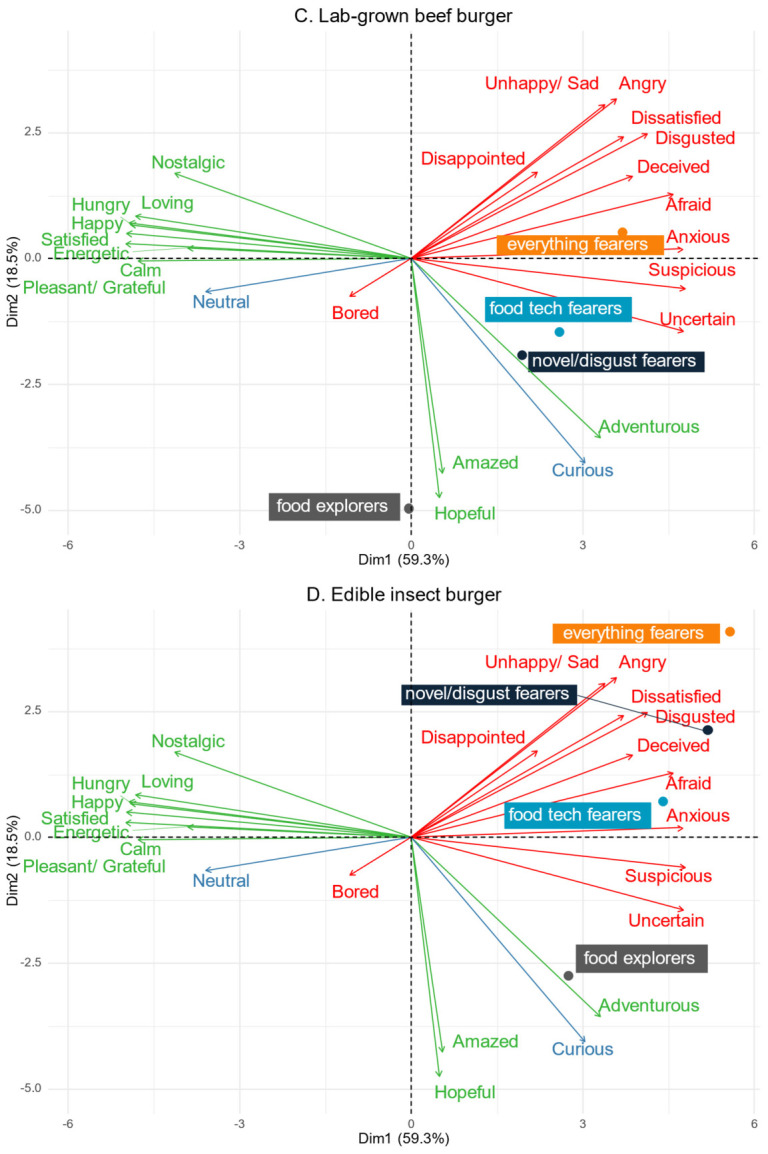
Principal component analysis biplot for four clusters of participants’ emotional responses for the burger patty types ((**A**): conventional beef; (**B**): plant-based; (**C**): lab-grown beef; and (**D**): edible insect). Note: All four graphs were generated in the same analysis. However, only one burger patty is shown at a time to help demonstrate better if/how the products are separated across the personal trait clusters. Emotions are classified into positive (*n* = 11, green), negative (*n* = 11, red), and unclassified (*n* = 2, blue) groups based on Orr, Giezenaar, Godfrey, and Hort [47].

Based on principal components analysis, emotional responses were found to vary by personal trait cluster across burger patty types, with 77.81% of the total variance explained. Dimension 1, which primarily captured differences in the valence of the emotions, accounted for 59.32%. Dimension 2 accounted for an additional 18.49%, and was mainly defined by the emotions *amazed*, *hopeful*, *curious*, and *adventurous *(Figure 6).

For the beef burger patty, emotional responses were largely consistent across all clusters, with participants in each cluster expecting predominantly positive emotions. This pattern suggests that conventional meat remains emotionally acceptable across different consumer segments (Figure 6A).

The emotional responses to the plant-based burger patty showed some variation between the personal trait clusters across Dimension 1 (Figure 6B). Notably, the food explorers were positioned closer to the positive emotions in the biplot. However, the everything fearers and novel/disgust fearers were positioned in the middle of the biplot, suggesting that very few emotions were evoked, and that for these clusters the plant-based burger patty mainly generated a neutral emotional reaction.

For the lab-grown beef burger patty, differences in emotional responses among the personal trait clusters were observed along both Dimensions 1 and 2 (Figure 6C). Dimension 1, which largely separated emotions based on valence, showed that everything fearers were more closely associated with negative emotions. In contrast, food explorers were positioned between the positive and negative emotions, indicating a more neutral emotional profile. Food explorers’ emotional responses were highly loaded onto Dimension 2, indicating that compared with the other personal trait clusters, they expected to be more *hopeful*, *amazed*, *curious*, and *adventurous* about lab-grown meat. This suggests a greater sense of perceived agency with the product among food explorers compared to the other clusters.

For the edible insect burger patty, only limited differences were found for Dimension 1, with all clusters positioned closer to the negative emotions (Figure 6D). However, the clusters were differentiated along Dimension 2, which may be related to dominance. Food explorers were more closely associated with *curious*, *amazed*, *hopeful*, and *adventurous*, particularly when compared to everything fearers. In contrast, everything fearers experienced stronger negative emotional responses toward the edible insect burger patty, including *dissatisfied*, *angry*, and *disgusted*. Taken together, these results suggest that the edible insect burger patty elicits broadly negative emotions across all personal trait clusters. Nonetheless, differences emerge in how these emotions are experienced. Food explorers may experience a greater sense of control and agency in their emotional responses, whereas everything fearers may perceive their responses as less controlled and more reactive. Food tech fearers and novel/disgust fearers had intermediate responses in relation to those of the other two groups. Nevertheless, Dimension 2 explained a relatively limited proportion of the total variance (18.5%); therefore, these results should be interpreted with caution.

Participants in all four personal trait clusters most frequently selected the conventional beef burger as the option they would purchase when forced to make a choice. Despite this, final burger patty choice was significantly associated with personal trait clusters (χ^2^(6) = 57.6, *p* < 0.001, Table 2), with a moderate effect size (Cramér’s V = 0.23). The conventional beef burger patty was selected more often than expected by chance by food tech fearers (*n* = 101, 71.1%) and everything fearers (*n* = 103, 80.5%). In contrast, food explorers were less likely than expected by chance to select the conventional beef burger patty (*n* = 49, 40.2%) and more likely to select the lab-grown beef burger patty (*n* = 38, 31.1%). Novel/disgust fearers selected the conventional beef burger in the expected proportion (60.4%), reflecting their status as the largest cluster. Nonetheless, this group selected the plant-based burger patty more often than expected by chance (*n* = 45, 24.7%). The edible insect burger patty was excluded from the Chi-square analysis because of low cell counts, which violated the assumptions of the test. This reflects its overall low selection rate (*n* = 14, 2.4%).

### 3.4. The Impact of Sharing Information on Consumer Responses to Protein Sources

**Figure 7:**
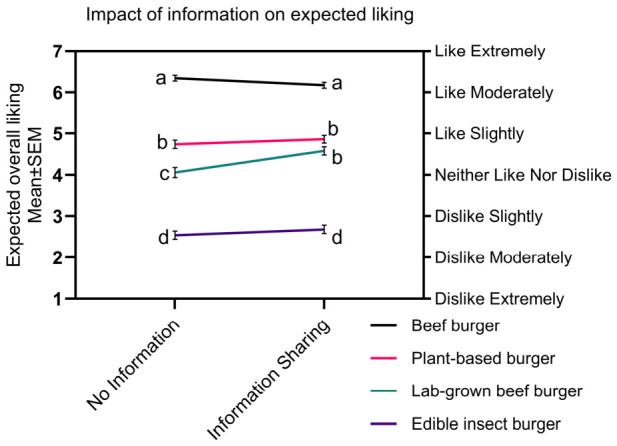
Interaction plot comparing the mean liking scores of participants in the ‘no information’ and ‘information sharing’ groups across the four burger patty types (conventional beef burger, plant-based burger, lab-grown beef burger, and edible insect). Significant differences in liking are indicated with different letters (*p* < 0.05, mixed-model ANOVA interaction, Tukey’s HSD).

A mixed-model ANOVA found a significant interaction between burger patty type and information sharing group (F(3,1716) = 5.1, *p* = 0.002, *η_p_*^2^ = 0.01). Participants that watched the video expected to like the lab-grown beef burger patty more (M = 4.58, 95% CI [4.38–4.79]) than participants that did not (M = 4.07, 95% CI [3.83–4.30]. However, no significant information sharing impact was found for the conventional beef, plant-based, or edible insect burger patties (Figure 7). Averaged across burger types, the main effect of information sharing group was approaching significance (F(1,1716) = 5.1, *p* = 0.061, *η_p_*^2^ < 0.01), suggesting that the video had very limited influence on liking in general. Consistent with the overall results, there was a significant main effect of burger patty type (F(3,1716) = 586.7, *p* < 0.001, *η_p_*^2^ = 0.51).

Information sharing was associated with differences in the expected final choice of burger patty (χ^2^(3) = 22.2, *p* < 0.001, Table 3), with a moderate effect size (Cramér’s V = 0.20). Compared with the information sharing condition, a higher proportion of participants in the no information condition selected the conventional beef burger patty than expected by chance. In contrast, the opposite pattern was observed for the lab-grown beef and plant-based burger patties, which were selected more frequently in the information sharing condition. No significant differences were observed in the selection of the edible insect burger patties between groups, likely reflecting its overall low selection rate.

## 4. Discussion

### 4.1. Overall Expected Liking and Emotional Responses to the Four Different Protein Sources

The strong preference for conventional beef observed in this study is consistent with previous research [39], indicating high levels of meat attachment among the sampled UK consumers [55]. Meat attachment reflects a deep emotional and habitual bond with meat consumption that develops through long-standing cultural norms and repeated consumption experiences. Meat attachment explained the strong preference for conventional beef and lower expectations for protein alternatives. As conventional meat is often perceived as nutritionally necessary and not environmentally damaging, it continues to function as the default and preferred protein choice for many consumers [56]. Although promoting a dietary shift towards protein transition is widely recognised as essential for achieving the United Nations Sustainable Development Goals, these findings highlighted a key challenge. Strong hedonic and habitual attachments to beef may act as a significant barrier to changing consumption patterns. Consistent with prior studies, plant-based and lab-grown meat were generally expected to be liked, whereas edible insects were associated with markedly lower acceptability [38,55,57,58,59].

The largely neutral and mildly negative emotional responses associated with plant-based burgers may reflect expectancy disconfirmation arising from prior consumption experiences. While plant-based burgers are widely available, previous studies indicated that they often fail to fully replicate the sensory properties of beef, particularly in juiciness and tenderness [60]. Repeated exposure to products that do not meet the sensory expectations of consumers may result in emotional responses characterised as dissatisfaction and disappointment, suggesting that consumers may have been bored or unsatisfied with the plant-based products available at the time of the survey. This implies that consumers’ neutral responses to plant-based meat may not stem from novelty or unfamiliarity, but from unmet expectations shaped by prior experience. Ford, Zhang, Gould, Danner, Bastian, and Yang [56] highlighted that taste is one of the most important motivating factors for consumers to adopt plant-based products. Therefore, optimising the taste and flavour of plant-based products is crucial for fostering more positive emotional responses to these products.

The participants in the study expected that a lab-grown beef burger patty would evoke both positive and negative emotional responses among consumers. The positive emotional responses may stem from consumers’ fascination with the innovative technology and its potential benefits (e.g., no animal slaughter, similar sensory properties to beef, more sustainable [21]). Despite its potential, some consumers are doubtful about lab-grown meat’s sustainability and raise concerns about its unnaturalness. Ford, Zhang, Gould, Danner, Bastian, and Yang [56] highlighted that UK consumers perceive lab-grown meat as unnatural, unnecessary, and unsustainable. Bryant and Barnett [17] also reported concerns that cultured meat is unhealthy or nutritionally inferior. Similarly, Weinrich et al. [61] noted that the emotional rejection of cultured meat is often driven by its perceived unnaturalness, consistent with the current study, where consumers selected the emotions *deceived* and *uncertain*. The heterogeneous emotional responses highlight the tension between the perceived innovation benefits and the psychological discomfort with food technology. Positive emotions reflect consumers’ sense of agency and interest in technological solutions, whereas negative emotions are likely to reflect concerns around unnaturalness. Although lab-grown meat is not yet available in the UK, lab-grown meat products have entered the markets in the US and Singapore [62]. The positive emotional responses observed in this study suggest market potential for lab-grown meat in the UK. However, addressing consumers’ negative emotional reactions and perceptions of unnaturalness will be critical for achieving widespread adoption.

In line with previous research [63,64,65,66], the strong negative emotions towards, low liking of, and extremely low willingness to choose edible insect burgers are consistent with evolutionary theories of disgust, according to which insects are commonly associated with contamination and threat in Western food systems [67]. Unlike plant-based or lab-grown meat, edible insects challenge deep cultural norms regarding what is considered appropriate food, particularly in Western societies where entomophagy lacks a historical basis. As a result, acceptance barriers for edible insects appear driven primarily by visceral emotional responses, such as disgust and fear, which could be hard to change.

This study provides preliminary insights into consumer attitudes towards meat alternatives, including products that are not yet commercially available in the UK. From a consumer behaviour perspective, expected liking and emotional responses represent anticipatory evaluations that are central to early attitude formulation and acceptance before consumers can taste the products. Such pre-consumption opinions play a critical role in shaping consumers’ initial responses to novel foods and their subsequent willingness to adopt them [68,69]. The heterogeneous emotional responses observed for lab-grown meat indicate clear market segmentation at the pre-market stage, reflecting diverse opinions and emotional associations. This pattern is consistent with previous research that has shown that novel food technologies often evoke mixed responses, where perceived benefits coexist with uncertainty or distrust [70]. However, anticipatory responses may change following direct sensory experiences, as expectations are either confirmed or disconfirmed after tasting [21,70,71]. Therefore, future research should examine consumers’ actual liking and emotional responses following sensory engagement with meat alternatives and integrate sensory evaluation methodologies to provide a more comprehensive understanding of novel food acceptance once the products are available for evaluation. Capturing consumers’ prior tasting or consumption experiences of protein alternatives would allow future studies to better account for the dynamic interplay between expectations, emotions, and subsequent consumer behaviour.

### 4.2. Impact of Personal Trait Clusters on Overall Liking

While previous research has examined food neophobia, food technology neophobia, and food disgust sensitivity largely as independent predictors of consumer responses to alternative proteins, their combined impact on consumer behaviour regarding protein alternatives has not been systematically studied [34,72,73,74]. By integrating these three traits using clustering, the present study advances the literature by identifying four distinct consumer segments that reflect the psychological pathways underlying acceptance or rejection of protein alternatives. This integrated approach highlights that barriers to novel protein acceptance are influenced by multidimensional factors including emotional, perceptual, and technology-related considerations.

The food explorers (21%) were characterised by lower scores for all three scales and demonstrated consistently higher levels of liking and more positive emotions towards all protein alternatives. From a psychological perspective, this level of openness suggests a lower sensitivity to perceived risk and greater motivation to engage with novelty. This cluster was the most closely associated with emotions such as *curiosity*, *satisfaction*, and *adventurousness*, which signals that they are more likely to value and are more open to unfamiliar products. This is evidenced by their significantly higher selection rate for the lab-grown beef burger patty in the hypothetical shopping scenario (31%), compared with 6–14% in the other clusters. Thus, they can be characterised as early adopters of new foods, indicating that food explorers may play a critical role in the initial uptake of novel protein products. Therefore, highlighting the novelty, sustainability, and technological advancements of protein alternatives might resonate particularly well with this group. Identifying such receptive groups emphasises the value of consumer segmentation in both academic research and commercial practice, as early adopters may act as catalysts for the wider social normalisation of protein alternatives.

The food tech fearers (25%) exhibited pronounced aversion towards novel food technologies, despite relatively low levels of food neophobia and food disgust sensitivity. Their particularly low acceptance of lab-grown meat supports that food technology neophobia is the primary barrier for this group. Rejection appears to be driven largely by scepticism towards products made with new technologies, which is often associated with concerns about safety, ethical issues, and perceived unnaturalness [27,30,75]. To effectively engage food tech fearers, strategies should prioritise reducing the perceived technological risks and uncertainty rather than introducing additional novelty. Importantly, a gradual adoption pathway may be considered, where familiar characteristics such as the taste and texture of the products are emphasised [76].

Novel/disgust fearers (32%) were characterised by high scores for both food neophobia and food disgust sensitivity, but relatively low food technology neophobia. Given that food disgust sensitivity and food neophobia are known to be correlated, the emergence of a cluster in which these traits co-occur is not unexpected. This indicates that their resistance is primarily driven by emotional responses to unfamiliar or disgust-eliciting foods, rather than novel technologies. Accordingly, this group showed particularly low acceptance of edible insects. This study indicates that consumers who have higher food disgust sensitivity are more likely to feel disgusted by edible insects and, therefore, reject edible insects as a food source, in line with Hartmann and Siegrist [32]’s research. Jensen and Lieberoth [63] noted that particularly in Western contexts, there is a deep-seated aversion to insects in food, especially when insect parts are visible, which is primarily a form of visceral disgust linked to contamination fear. Considering the potential strategies for novel/disgust fearers, introducing the novel protein alternatives in a familiar or culturally normalised product format and context might help mitigate fear and unfamiliarity.

Everything fearers (22%) were characterised by simultaneously high scores for all three food-related avoidance scales, indicating a broad aversion to novel food, new production technologies, and disgust-eliciting products. They were the cluster that was most likely to select the conventional beef burger patty at the end of the hypothetical shopping scenario (81% compared with 40% of food explorers, 71% of food tech fearers, and 60% of novel/disgust fearers). Furthermore, compared with food explorers, everything fearers had significantly lower liking for the plant-based, lab-grown, and edible insect burger patties, alongside stronger negative emotional responses. This emotional aversion may play a crucial role in shaping resistance, making this group the least receptive consumer segment for protein alternatives. Given the substantial psychological barriers observed in this group, further research should explore whether hybrid products that combine conventional meat with protein alternatives offer a more acceptable pathway towards gradual adoption.

This is the first study to characterise consumer responses across the four distinct personal trait clusters, revealing clear differences in expected responses to protein alternatives. These findings highlight the importance of identifying personal-trait-based consumer segmentation for developing targeted engagement and behavioural change strategies in real-world applications. For example, food explorers appear to be the most promising target group for promoting alternative proteins. Novel/disgust fearers show selective potential for lab-grown meat. These two consumer clusters could be the potential target consumer groups for lab-grown meat, as they are more open to novel or technology-driven products. In contrast, everything fearers were consistently resistant. Additionally, no significant differences in age, gender, or education level were observed between the clusters, suggesting that the identified segmentation was not driven by sociodemographic factors. However, the results should be interpreted with caution in relation to the wider UK population, and further research using larger, representative samples is required to assess the robustness and generalisability of these clusters. Importantly, this would allow for the incorporation of sociodemographic variables as potential mediating factors, which may provide additional insights into the relationships between personal traits and consumer responses.

### 4.3. Impact of Information on Expected Liking

Consumers in the information sharing condition selected meat alternatives more frequently (46%) than those in the no information condition (27%), with significant shifts observed for both the plant-based and lab-grown beef burger patties. However, this broader effect on choice behaviour contrasted with liking responses, where only the lab-grown beef burger patty showed a significant change following information provision. This divergence suggests that while participants’ expected liking remained largely unchanged, their expected willingness to try alternative protein products increased following exposure to information [77]. Given that liking is typically driven by anticipated sensory qualities such as taste, texture, and appearance [78], this pattern suggests that the provision of environmental information may encourage consumers to make trade-offs that influence choice without necessarily altering hedonic expectations. Further research is needed to determine whether these effects on choice persist over time or primarily reflect short-term responses immediately following information exposure [79].

### 4.4. Limitations

Several limitations of this study should be acknowledged. First, the sample was recruited using a convenience sampling approach and was skewed towards younger participants and females. As a result, the findings may not accurately reflect the wider UK population. Instead, the results provide exploratory insights into patterns of consumer responses within the sampled group and should be interpreted as indicative. In addition, only meat eaters were included in the study. As meat eaters and non-meat eaters differ in their preferences and expectations of meat alternatives, the perspectives of non-meat-eating consumer groups were not captured and therefore cannot be generalised from the present findings [80]. Further research using a representative sampling design is therefore needed to confirm these findings across the broader UK population.

Second, the observed increase in expected liking and selection of lab-grown meat in the information sharing condition may have been partly driven by the specific video content. The video combined environmental messaging with an explanation of its production process. In contrast, the production processes for the other burger patties were not included. Given the relatively low public familiarity with lab-grown meat, this additional information may have reduced uncertainty leading to a disproportionate increase in the positive response towards this product only. Therefore, the information sharing results are presented as exploratory.

Third, the study did not capture potentially relevant control variables, including prior experience with alternative proteins, income, lifestyle factors, broader food consumption habits, or the regional distribution of participants. These factors may influence expectations of novel protein sources and should be considered in future research.

Fourth, the use of hypothetical online shopping scenarios may have limited ecological validity. For example, in a study comparing plant-based and pork hot dogs [81], no significant differences between the products were observed in willingness to buy or expected liking prior to tasting. However, following consumption, the plant-based hot dog failed to meet consumers’ sensory expectations, leading to negative disconfirmation, whereby both liking and willingness to buy decreased. In contrast, the pork hot dog met or exceeded sensory expectations, resulting in increased liking and willingness to buy. As plant-based meat alternatives often fail to meet consumers’ sensory expectations [82], it remains unclear if/how the results would be different if the participants had had the opportunity to cook and taste the burger patties. While plant-based burgers are readily available in the UK, insect-based and lab-grown beef burger patties are not. Consequently, consumers’ expectations of these products are likely to be less grounded in prior experience, potentially resulting in a larger gap between expectations and actual sensory experience. In addition, while the use of a hypothetical shopping scenario may help participants to contextualise and imagine a shopping experience, it does not fully reflect real-world decision making. In real settings, food choice is influenced by many factors (e.g., price, cultural norms, knowledge/skills, access, and habits [83]), and there are consequences for making a bad choice (e.g., food waste or negative experience [84]). Thus, the results of this study may also be impacted by hypothetical bias, which is defined as the discrepancy between responses collected in hypothetical and realistic settings [85]. Caution should be applied when interpreting the choices, liking, and emotional responses reported here, as they reflect anticipatory judgements rather than real evaluations. Further research is needed to validate these findings in more ecologically valid settings, where consumers can purchase, prepare, and consume products repeatedly over time, allowing for the assessment of how expectations, emotions, and acceptance evolve over repeated exposure.

## 5. Conclusions

As global concerns over sustainable food systems rise, this study confirmed that UK consumers still show a strong preference for conventional beef burgers compared with other protein alternatives, albeit in a convenience sample. This indicates that consumer acceptance of alternative proteins remains constrained, and multiple approaches and interventions are needed to promote the adoption of alternative proteins. Using a lexicon developed specifically for meat alternatives, distinct emotional profiles were identified for each protein source: conventional beef elicited predominantly positive emotions; edible insects evoked strong negative emotions; lab-grown beef elicited a mix of positive and negative emotions; and plant-based options were associated with largely neutral emotions. Based on food-related avoidance traits (FNS, FTNS, and FDS), four preliminary consumer clusters were identified, each exhibiting different response patterns to the protein sources. Food explorers appeared to be the most likely early adopters of protein alternatives, as they scored low for all the personal traits and have the highest acceptance levels of all the protein sources. In contrast, everything fearers scored high for all the traits and exhibited the lowest acceptance among the clusters. Thus, this cluster is likely to face substantial psychological barriers to the adoption of protein alternatives. Novel/disgust fearers and food tech fearers were also identified, and their responses were between these two extremes. These findings highlight the value of consumer segmentation for informing more targeted marketing and product development strategies, suggesting that different groups may require distinct messages or product formats at the pre-market stage. However, this study focuses on expected responses. Therefore, further research incorporating sensory exposure and longitudinal design is needed to capture how expected liking, emotional responses, and choices may change with tasting experience and repeated exposure, both of which are critical for understanding real-world behaviour and market adoption.

## Figures and Tables

**Table 1:** Summary of names, images, and descriptions of the four burger patty types.

Burger Patty Type	Image	Description
Conventional beef burger patty	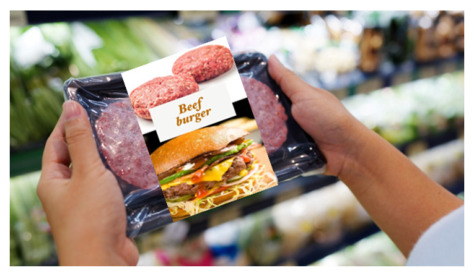	This beef burger patty is made from meat from cows that were raised by conventional methods on a farm.
Plant-based burger patty	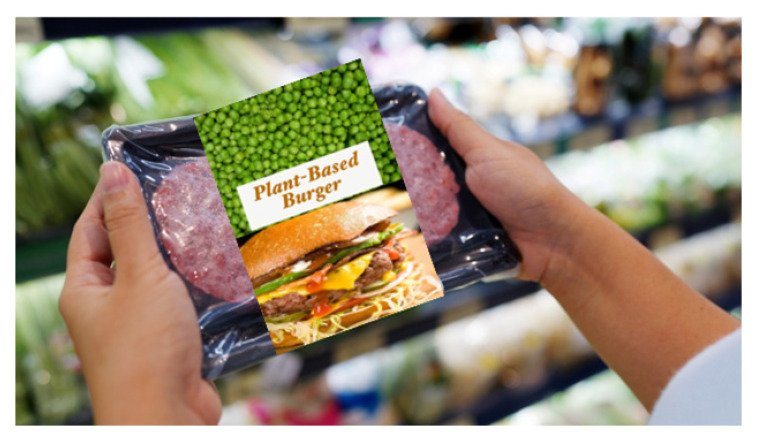	This plant-based patty is made with peas, with no animal products.
Lab-grown beef burger patty	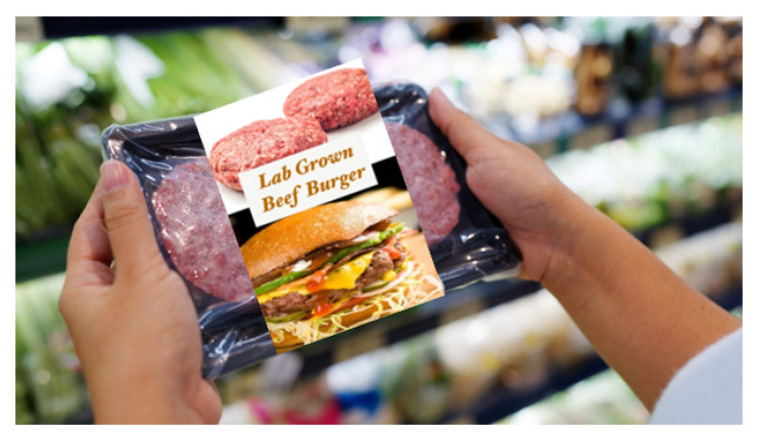	This lab-grown beef patty is produced using animal cells that are taken from living cows. The cells are fed animal-free nutrients and transferred to fermentation tanks, which allow them to grow into muscle strands before being formed into a burger.
Edible insect burger patty	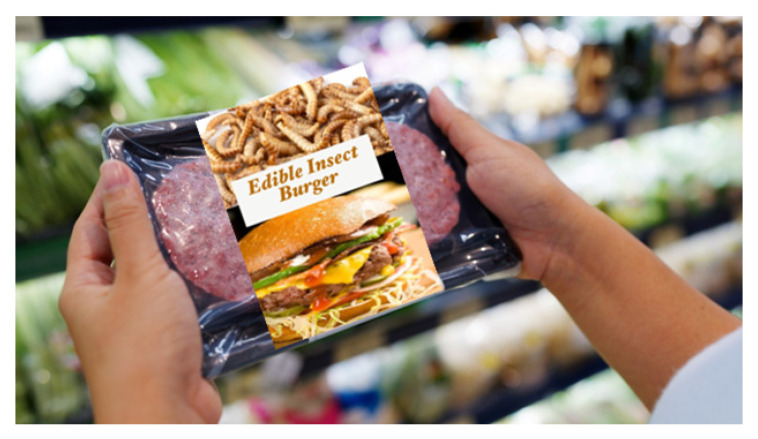	This edible insect burger patty is made from buffalo worms, blended with plant-based ingredients.

**Table 2:** Chi-square contingency analysis of the final burger patty choice by personal trait cluster. Cells marked with (+) or (−) indicate observed frequencies higher or lower than expected, respectively. ^#^ The edible insect burger category was excluded from the contingency analysis due to low cell counts.

Burger Patty Choice	Food Explorers (*n* = 122, 21%)	Food Tech Fearers (*n* = 142, 25%)	Novel/Disgust Fearers (*n* = 182, 32%)	Everything Fearers (*n* = 128, 22%)
Conventional beef (*n* = 363, 63.2%)	49 −	101 +	110	103 +
Plant-based (*n* = 111, 19.3%)	29	20	45 +	17 −
Lab-grown beef (*n* = 86, 15.0%)	38 +	15	26	7 −
Edible insect (*n* = 14, 2.4%)	6 ^#^	6 ^#^	1 ^#^	1 ^#^

**Table 3:** Chi-square contingency analysis of the final burger patty choice information sharing condition. Cells marked with (+) or (−) indicate observed frequencies higher or lower than expected, respectively.

Burger Patty Choice	No Information (*n* = 277, 48.3%)	Information Sharing (*n* = 297, 51.7%)
Conventional beef (*n* = 363, 63.2%)	202 +	161 −
Plant-based (*n* = 111, 19.3%)	42 −	69 +
Lab-grown beef (*n* = 86, 15.0%)	29 −	57 +
Edible insect (*n* = 14, 2.4%)	4	10

## Data Availability

The original contributions presented in this study are included in the article. Further inquiries can be directed to the corresponding author.

## Abbreviations

FNS	Food Neophobia Scale
FTNS	Food Technology Scale
FDS	Food Disgust Scale

## Appendix A

**Table A1:** Personal traits and age by cluster. Different letters indicate significant differences (one-way ANOVA).

Cluster	Mean [95% CI]				Statistics
	Food Explorers (*n* = 122, 21%)	Food Tech Fearers (*n* = 142, 25%)	Novel/Disgust Fearers (*n* = 182, 32%)	Everything Fearers (*n* = 128, 22%)	
Personal traits					
FNS (Potential range 10–70)	17.8 D [16.9–18.6]	21.8 C [20.5–23.1]	30.0 B [28.9–31.1]	39.0 A [37.6–40.4]	F = 216.1 *p* < 0.0001 *η_p_*^2^ = 0.53
FTNS (Potential range 13–91)	41.9 D [40.7–43.1]	58.5 B [56.7–60.3]	50.3 C [49.2–51.3]	62.3 A [61.0–63.5]	F = 158.0 *p* < 0.0001 *η_p_*^2^ = 0.45
FDS (Potential range 7–42)	21.7 C [20.9–22.5]	17.6 D [17.0–18.3]	28.3 B [27.6–29.0]	30.0 A [29.0–30.9]	F = 219.7 *p* < 0.0001 *η_p_*^2^ = 0.54
Age					
Age (Range 18–81)	31.8 [29.7–33.8]	33.9 [31.7–36.0]	34.3 [32.5–36.1]	34.1 [32.2–36.1]	F = 1.3 *p* = 0.287 *η_p_*^2^ < 0.01

**Table A2:** Demographic differences between the clusters (Chi-square). Post-hoc results are shown with “+” and “−”, which indicate a higher or lower than chance percentage of participants for a given group, respectively. ^#^ The ‘prefer not to say’ and ‘other’ categories were excluded from the contingency analysis due to low cell counts.

Cluster	Food Explorers (*n* = 122, 21%)	Food Tech Fearers (*n* = 142, 25%)	Novel/Disgust Fearers (*n* = 182, 32%)	Everything Fearers (*n* = 128, 22%)	Statistics
Gender					
Female (*n* = 408, 71.1%)	78	104	138	88	χ^2^(3) = 5.7 *p* = 0.128 Cramér’s V = 0.10
Male (*n* = 166, 28.9%)	44	38	44	40	
Other ^#^	0	0	0	0	
Prefer not to say ^#^	0	0	0	0	
Education					
Secondary school (*n* = 78, 13.6%)	11	25	22	20	χ^2^(9) = 13.4 *p* = 0.147 Cramér’s V = 0.09
College diploma or certificate (*n* = 116, 20.2%)	20	28	41	27	
Undergraduate university degree (*n* = 231, 40.2%)	47	60	71	53	
Postgraduate university degree (*n* = 148, 25.8%)	44	29	47	28	
Prefer not to say ^#^	0 ^#^	0 ^#^	1 ^#^	0 ^#^	
Diet					
Flexitarian (*n* = 41, 7.1%)	6	7	23 +	5	χ^2^(3) = 12.2 *p* = 0.007 Cramér’s V = 0.15
Omnivore (*n* = −533, 92.9%)	116	135	159 −	123	

**Table A3:** Summary of the overall citation proportions for the emotion term and corresponding Cochran’s Q test results. To correct for multiple comparisons (*n* = 24), Bonferroni correction was used to calculate an adjusted alpha of 0.021 when testing for significance. Products that differ significantly in citation proportions are marked with different letters (critical difference, Sheskin procedure). Emotions are classified into positive, negative, and unclassified groups based on Orr, Giezenaar, Godfrey, and Hort [47]. Citation proportions above 0.1 are coloured grey, where higher citation proportions are a darker shade.

Emotion	Citation Proportion				*p*-Value
	Conventional Beef Burger Patty	Plant-Based Burger Patty	Lab-Grown Beef Burger Patty	Edible Insect Burger Patty	
Positive emotions					
Calm	0.251 (b)	0.202 (b)	0.064 (a)	0.026 (a)	<0.0001
Adventurous	0.023 (a)	0.146 (b)	0.303 (c)	0.277 (c)	<0.0001
Energetic	0.101 (b)	0.037 (a)	0.031 (a)	0.026 (a)	<0.0001
Pleasant/ Grateful	0.451 (d)	0.220 (c)	0.108 (b)	0.042 (a)	<0.0001
Loving	0.120 (c)	0.044 (b)	0.017 (ab)	0.005 (a)	<0.0001
Amazed	0.068 (a)	0.066 (a)	0.172 (b)	0.078 (a)	<0.0001
Hopeful	0.085 (a)	0.260 (b)	0.268 (b)	0.131 (a)	<0.0001
Happy	0.549 (d)	0.211 (c)	0.108 (b)	0.023 (a)	<0.0001
Hungry	0.406 (d)	0.225 (c)	0.141 (b)	0.066 (a)	<0.0001
Nostalgic	0.150 (b)	0.010 (a)	0.014 (a)	0.010 (a)	<0.0001
Satisfied	0.692 (d)	0.282 (c)	0.159 (b)	0.033 (a)	<0.0001
Negative emotions					
Unhappy/ Sad	0.040 (a)	0.047 (a)	0.051 (a)	0.141 (b)	<0.0001
Dissatisfied	0.030 (a)	0.169 (c)	0.089 (b)	0.226 (d)	<0.0001
Angry	0.010 (a)	0.009 (a)	0.024 (a)	0.054 (b)	<0.0001
Anxious	0.024 (a)	0.047 (a)	0.228 (b)	0.319 (c)	<0.0001
Afraid	0.005 (a)	0.023 (a)	0.136 (b)	0.280 (c)	<0.0001
Suspicious	0.010 (a)	0.124 (b)	0.418 (c)	0.470 (c)	<0.0001
Bored	0.012 (a)	0.077 (b)	0.009 (a)	0.005 (a)	<0.0001
Disappointed	0.033 (a)	0.166 (c)	0.064 (a)	0.115 (b)	<0.0001
Deceived	0.007 (a)	0.047 (b)	0.061 (b)	0.066 (b)	<0.0001
Disgusted	0.026 (a)	0.042 (a)	0.136 (b)	0.530 (c)	<0.0001
Uncertain	0.024 (a)	0.263 (b)	0.477 (c)	0.488 (c)	<0.0001
Unclassified emotions					
Curious	0.028 (a)	0.383 (b)	0.544 (c)	0.392 (b)	<0.0001
Neutral	0.209 (c)	0.284 (d)	0.127 (b)	0.045 (a)	<0.0001
